# Prototyping as subtyping strategy for studying heterogeneity in autism

**DOI:** 10.1002/aur.2535

**Published:** 2021-06-02

**Authors:** Michael V. Lombardo

**Affiliations:** ^1^ Laboratory for Autism and Neurodevelopmental Disorders Center for Neuroscience and Cognitive Systems @UniTn, Istituto Italiano di Tecnologia Rovereto Italy; ^2^ Autism Research Centre, Department of Psychiatry University of Cambridge Cambridge UK

“*All models are wrong but some are useful*” (Box, 1979, p. 202). This statement is particularly useful when it comes to the topic of studying of heterogeneity in autism. Expanding on ideas initially presented in Mottron and Bzdok (2020), Mottron's (2021) new commentary in this journal explicitly calls out how the current model for how we study autism is wrong and that there are other models (i.e., “prototypical autism”) that may be more useful. I think the field would resoundingly agree that the current model, resting on blind usage of the single diagnostic label, is less than ideal when it comes to furthering scientific research. Happé et al.'s (2006) paper was certainly one of the first to avert my attention to the issue. Many subsequent papers have come since then elaborating on the idea that heterogeneity in autism is a central scientific challenge for the field and also to discuss ways to address this challenge (e.g., Geschwind & Levitt, 2007; Gillberg, 2010; Lai et al., 2013; Lombardo et al., 2019; London, 2007; Müller & Amaral, 2017; Waterhouse & Gillberg, 2014).

**FIGURE 1:**
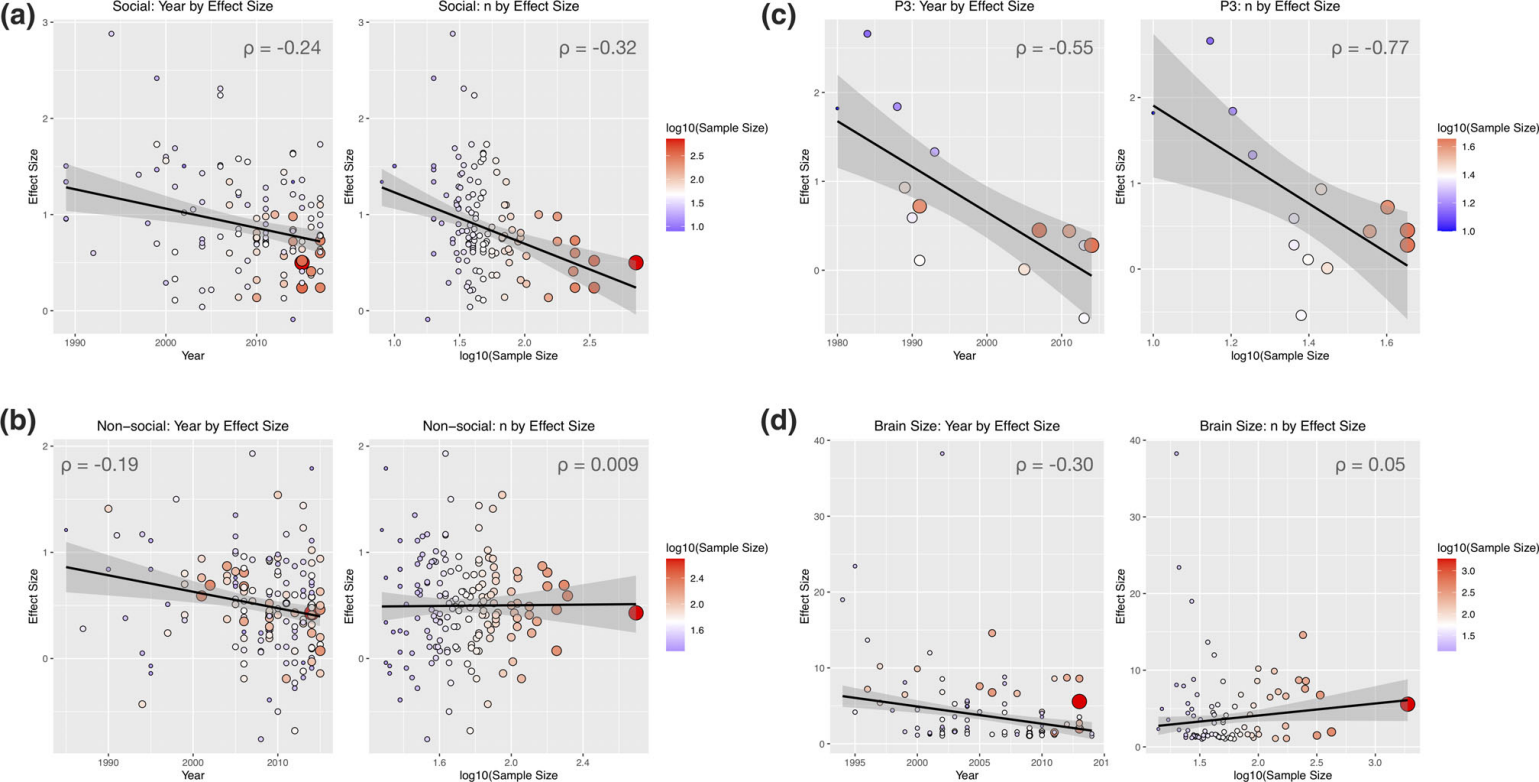
Relationships between time, sample size, and effect size in autism research (a re‐analysis of data from Rødgaard et al., 2019). Panel (a) shows relationships for studies classified as social cognition (e.g., emotion recognition and theory of mind). Panel (b) shows the same plots for studies of non‐social cognitive tasks (e.g., flexibility, planning, inhibition). Panel (c) shows plots for studies of the P3 amplitude, while Panel (d) shows plots for studies of brain size. In each plot, each study is a single dot and Spearman's *ρ* is shown to describe the relationship. The coloring of the dots reflects the log10(sample size) (hotter colors reflecting larger sample size studies). The size of each study's dot also changes according to sample size (bigger reflecting larger sample size). The linear best‐fit line is also shown, and this best fit is estimated with robust linear modeling in order to be insensitive to outliers. Reproducible data and analysis code for this re‐analysis can be found here: https://github.com/IIT‐LAND/autism_es_time_n

While we would agree that the single diagnostic label is not ideal, we likely disagree somewhat with how a concept like “prototypical autism” might be an important alternative to the current situation. By studying prototypical autism, Mottron (2021) suggests we would reap the benefits of much larger effect sizes and thus gain more clarity in terms of a mechanistic understanding of autism. The claim of a bigger effect sizes as one goes further back into the past (Rødgaard et al., 2019) is not without some problems though. Sample size in autism research has also increased over time, and it is well known that effect sizes can be inflated (and sometimes by very large margins) in situations with small sample sizes (see simulations within Lombardo et al., 2019). In Figure 1, I have re‐plotted data from Rødgaard et al. (2019), to show both the year‐by‐effect size and sample size‐by‐effect size relationships, with Spearman's ρ annotated on each plot. For the reproducible data and code for this re‐analysis, see here: https://github.com/IIT-LAND/autism_es_time_n. For studies of social cognition and the P3b amplitude, both relationships between year and effect size, and sample size and effect size are present. Also notable is the decrease in variance of effect size as sample size increases, showing the well understood phenomenon that as sample size increase, the precision of the estimated statistic increases and likely converges on what the true population parameter is likely to be. This effect alone underscores the primary importance of larger sample sizes. Since effect size inflation is also a known issue (Lombardo et al., 2019), it is unclear whether higher effect sizes in the past are indeed precise estimates, because effect sizes are more imprecise with smaller sample sizes and older studies have much smaller sample sizes than studies of today (i.e., in all cases, except studies of non‐social cognitive tasks, sample size and year are also significantly positively correlated). Trying to statistically covary out sample size, as Rødgaard et al. (2019) did is not really the best remedy for this situation, since removing variation associated with sample size also removes shared variance for year, due to the fact that year and sample size are positively correlated.

However, let us assume that it is the case that studying prototypical autism results in larger effect sizes. There is still a primary conceptual issue at the heart of this argument. Mottron's view of prototypical autism squarely dismisses ideas about heterogeneity, diversity, and individual differences as being nothing more than artifact—in fact, Mottron and Bzdok (2020) declare this take‐home message unequivocally in the title of their paper: “Autism spectrum heterogeneity: fact or artifact”. Mottron's view implies that autism is best conceived of as singular unitary entity and not as a spectrum with important individual differences. The success of this kind of prototypical autism model depends somewhat on whether this underlying assumption is indeed valid and more correct than other models that would not assume that autism is one singular entity.

Besides this issue, I can see other problems with applying Mottron's approach in practice. First, what is prototypical autism? Mottron suggests that prototypical autism is a distinction that is clear to clinical experts, but it is never properly given a clear definition. Second, amongst the multiple levels one could examine in autism, the proposition of studying prototypical autism is one that places priority or importance at levels like behavior, cognition, and the clinical phenotype. What about other models that instead look for genetic or neural distinctions (e.g., Hong et al., 2020)? Why would the approach of looking at what is “prototypical” about autism at cognitive, behavioral, or clinical levels be more important than a definition of what is “prototypical” or most common about autism at other more biological levels of analysis?

A hardliner approach would be to declare that studying Mottron's “prototypical autism” as the best and only way to move forward. Taking this hardliner route is probably equally as wrong as the current situation. I would rather view what Mottron proposes in a slightly different light and also as just one of many different types of possible approaches for understanding heterogeneity in autism. Focusing on “prototypical autism” is just one of many possible “*supervised*” models for studying heterogeneity in autism (for more on “*supervised*” vs. “*unsupervised*” approaches to studying heterogeneity in autism, please see Lombardo et al., 2019). With the right set of justifications and the explicit end goals pre‐specified, along with nuanced interpretation and care to not overgeneralize, there is no reason why studying “prototypical autism” could not yield insights for the field.

In terms of how to construe what is: prototypical,” we could benefit by looking at analogies taken from other approaches. For example, normative modeling approaches (Bethlehem et al., 2020; Marquand et al., 2016) are one of many ways to study heterogeneity and are well suited for detecting outlier individuals that markedly differ from age‐appropriate norms defined from a non‐autistic comparison population. With normative modeling, one first defines what the non‐autistic comparison population norms are, and then autistic individuals are described statistically relative to those norms. The concept of “prototypical autism” seems somewhat analogous with respect to idea that first, a norm or prototype needs to be defined, and this would help make the definition of the “prototype” a bit more objective. However, the population that the norm is estimated on would need to be the autism population itself. If one could define the degree to which an autistic individual conforms to the autism norm or prototype, a whole range of phenomena could then be studied with respect to how individuals may deviate from that prototype. An example of how this can be done comes from our recent work where we characterized the joint distribution of social‐communication (SC) and restricted repetitive behavior scores from the autism population and then used those norms to create subgroups (Bertelsen et al., 2021). An advantage to construing the idea of “prototypical autism” within this kind of “normative modeling” framework is the allowance for the norm to be defined on the basis of any collection of measured variables, which could come from any level of analysis. Furthermore, what is considered the definition of the norm or prototype is now empirically defined in statistical terms (e.g., a z‐score based on the mean and SD of the autism population). The approach also does not a priori place higher importance on a few phenotypic levels (e.g., behavior, cognition) and could be implemented on any set of variables characterizing the autism population (e.g., cortical thickness phenotypes derived from structural MRI). Thus, rather than rejecting the idea of heterogeneity and assuming autism is one thing, this approach could take the concept of “prototypical autism,” formally define it based on empirically‐derived statistical norms from the autism population, and then allow researchers to select and restrict their samples accordingly. The inferences one could generate from this approach would then need to be specific to the subset of the population and the set of variables that originally defined by the norm or prototype under study. In this way, the model's usefulness is preset based on a priori supervised justifications for how and why the researcher first defines the prototype under study. The model may not be panacea to all problems we face, and thus it may be wrong in many respects, but at least it would be explicitly clear from the start what it is useful for.

In sum, a view that interprets prototypical autism to be one of many possible strategies for dealing with heterogeneity in autism could reap significant insights if applied in a nuanced way, where the goals and justifications are clearly spelled out, the disadvantages are clearly understood, and the interpretations are not over‐ or under‐generalized. There is room for many possible models. All will likely be wrong in some way, but it is very possible that different models are useful in different ways. We should exploit all possible models for their unique utility until better models come along that are empirically demonstrated to be less wrong.
